# Evaluation of Fresh Water Actinomycete Bioflocculant and Its Biotechnological Applications in Wastewaters Treatment and Removal of Heavy Metals

**DOI:** 10.3390/ijerph16183337

**Published:** 2019-09-10

**Authors:** Mayowa Oladele Agunbiade, Carolina Pohl, Esta Van Heerden, Oluwaseun Oyekola, Anofi Ashafa

**Affiliations:** 1 Biocatalysis and Technical Biology Research Group, Cape Peninsula University of Technology, PO Box 1906, Bellville 7535, South Africa; 2 Department of Microbial, Biochemical and Food Biotechnology, University of the Free State, Bloemfontein 9301, South Africa; 3 iWater Pty Limited, Walter Sisulu 5, Bloemfontein 9301, South Africa; 4 Department of Chemical Engineering, Cape Peninsula University of Technology, Bellville 7535, South Africa; 5 Phytomedicine and Phytopharmacology Research group, Department of Plant Sciences, University of the Free Sate, Qwaqwa Campus, Puthaditjabha 9866, South Africa

**Keywords:** wastewaters, *Terrabacter* sp, flocculation, heavy metals, ICP-OES, FTIR

## Abstract

This study evaluated the potential of a biopolymeric flocculant produced by *Terrabacter* sp. isolated from Sterkfontein Dam, South Africa. Microbial flocculants aid the aggregation of suspended solutes in solutions, thus, suggesting its alternative application to inorganic and synthetic organic flocculants, which are associated with health-related problems. The 16S rDNA analysis revealed the bacteria to have 98% similarity to *Terrabacter* sp. MUSC78T and the sequence was deposited in the Genbank as *Terrabacter* sp. with accession number KF682157.1. A series of experimental parameters such as bioflocculant dosage, cations concentrations, pH, and application of the purified bioflocculant in wastewaters treatment were investigated. In the presence of glucose as a sole carbon source, Ca^2+^ as cation at pH 8, the optimal flocculating activity attained was 85%. Optimum bioflocculant dosage of 0.5 mg/mL was able to remove chemical oxygen demand (COD), biological oxygen demand (BOD), suspended solids (SS), nitrate, and turbidity in dairy wastewater. In addition, the tested bioflocculant exhibited higher flocculating efficiency as compared to polyaluminum chloride, polyethylenime, and alum. Inductible coupled plasma optical emission spectroscopy (ICP-OES) analyses confirmed significant removal of 77.7% Fe, 74.8% Al, 61.9% Mn, and 57.6% Zn as representatives of heavy metals from treated dairy wastewater. Fourier transform infrared spectroscopy (FTIR) indicated the presence of carboxyl, hydroxyl, and amino groups in the purified bioflocculant which could be responsible for flocculation. Findings from this study showed the prospect of the studied bioflocculant as an alternative candidate in wastewater treatment and remediating of heavy metals.

## 1. Introduction

Increase in industrialization and anthropogenic activities has resulted in tremendous increases in the discharge of wastes and wastewater containing organic and inorganic pollutants into the environment [1]. Wastewaters containing heavy metals are directly or indirectly discharged into the environment, and consequently pose a serious threat to public health. Cadmium, lead, and mercury are the most dangerous of these metals and they are known as toxic-trio metals. Murthy [2] reported that toxic metals adversely affect physiological function by blocking the active sites of enzymes, displacing the major metal ions and altering the conformation of proteins.

Coagulation-flocculation technology is widely used with the aid of flocculants in the treatment of wastewaters, especially in the removal of suspended solids, particles, and debris [3]. Chemical flocculants such as polyaluminum chloride, polyethyleneime, aluminum sulfate, and ferric chloride have been used in water treatment. However, they are not biodegradable and therefore not environmentally friendly. In addition, aluminum salts and polyacrylamide derivatives have been reported to be associated with health problems such as Alzheimer’s disease and cancer, respectively [4,5]. Therefore, using both the inorganic and synthetic organic flocculants could aggravate environmental and health concerns. Hence, the development of environment-friendly biological macromolecules flocculants have become inevitability. Previous studies have revealed that algae, fungi, bacteria, and actinomycetes as a producer of flocculants that could be used as substitutes for chemical flocculants in wastewater treatment [6,7]. Lately, bioflocculants have attracted wide attention due to their biodegradability and safety in wastewater treatment, treatment of dye solutions, inorganic solid suspensions, downstream processing, meat processing wastewater, and removal of heavy metals [8,9,10]. Microbial flocculants are metabolites of microorganisms that are composed of high molecular weight compounds such as polysaccharides, proteins, glycoproteins, and proteoglycans [11]. Bacteria produce range of exopolysaccharides which are synthesized through different biosynthesis pathways that play a vital role in formation of biofilm and biosorption of metals [12,13]. Extracellular metabolites produced during cell lysis could act as energy sources for microbes during starvation and mediate in cell to cell interactions, thereby enhancing the adherence of cells to the surface [14].

Although, certain strains of actinobacteria have been implicated in flocculation, their industrial application on a large-scale medium is yet to be explored. Hence, there is need to explore more actinobacteria strains with enhanced flocculation efficiency to serve as a replacement or substitute for chemical synthesized flocculants. In this present study, we reported on the isolation, screening, and characterization of a bioflocculant produced by the actinomycete, *Terrabacter* sp. and its biotechnological application in wastewaters treatment and heavy metal removal.

## 2. Materials and Methods

### 2.1. Sample Collection

Soil sediment and water samples were collected aseptically from the site using sterile containers and air-tight bottles. Sample bottles and containers were stored in a cooler box containing ice-pack. The samples were processed within 12 h on arrival in the laboratory.

### 2.2. Cultivation and Isolation of Bioflocculant-Producing Actinomycetes

Twenty grams (20 g) of each sediment sample was air-dried at room temperature for two to three days, crushed, sieved through a 2 cm mesh, and kept refrigerated (4 °C) until use. Briefly, about 5 g was used from the air-dried sample and serial dilutions with sterile distilled water were carried out. The method of Jensen [15] was adopted with little modifications in the cultivation of samples and isolation of actinomycetes strains by using yeast malt extract agar (YME). The isolation agar (YME) was supplemented with 50 mg/L cyclohexamide and 20 mg/L nalidixic acid to minimize the growth of other bacteria and fungi. After culturing, strains of actinomycetes colonies (specific size, shape, aerial mycelium, and color) were further purified on YME plates and stored in the fridge at 4 °C

### 2.3. Screening for Bioflocculant-Producing Actinomycetes

The protocol of Xia et al. [16] with little modification was employed in the screening of four isolated actinobacteria strains. The isolates were inoculated into bottles containing 5 mL screening composition medium of 2% glucose, 0.05% urea, 0.05% yeast extract, 0.02% (NH_4_)_2_SO_4_, 0.01% NaCl, 0.02% MgSO_4_.7H_2_O, 0.5% K_2_HPO_4_, and 0.2% KH_2_PO_4_ in distilled water. The cultures were incubated for 48 h on a rotatory shaker at 30 °C, 160 rpm at pH 7. This pre-culture was used as the standard inoculum preparation for further experiment. Furthermore, 100 µL (pre-culture) was inoculated into production medium (1% glucose, 0.1% peptone, 0.03% MgSO_4_.7H_2_O, 0.5% K_2_HPO_4_, and 0.2% KH_2_PO_4_ in distilled water) and incubated at 25 °C in a rotatory shaker (Inco shake, Labotec) at 130 rpm for 48 h at pH 7. After incubation, 2mL of the broth was withdrawn and centrifuged at 3000 rpm for 30 min at 15 °C. The cell-free culture supernatants were collected and used to evaluate the efficiency of bioflocculant produced against kaolin clay suspension, a test material to simulate the turbidity of a surface water. The positive strain that exhibited the best flocculating activity was chosen for further analysis. Furthermore, the flocculating efficiency of actinomycetes strains were determined using kaolin clay suspensions as previously performed by Agunbiade [17]. Kaolin suspension was prepared by dissolving 4 g of kaolin clay in 1 L distilled water. A volume of 100 mL kaolin suspension was added to 3 mL of calcium chloride (1% *w*/*v*) and 2 mL of cell-free supernatant in 250 mL conical flask. The mixture was thoroughly shaken for 30 s at room temperature, gently poured into 100 mL measuring cylinder and allowed to settle for 5 min. The optical density (OD) of the upper solution was measured using a spectrophotometer (UV/Visible Biowave II and Biowave II, + England) at 550 nm. The control experiment was carried out in the same way, but cell-free supernatant was replaced with 2 mL of freshly prepared production medium. The flocculating activity (FA) was calculated using the equation:

(1) $$FA\left(\%\right)=\frac{B-A}{B}\times 100$$

where: A and B are the respective absorbance of the sample and control experiment at 550 nm.

### 2.4. Identification of Organism

Using the ZR Fungal/Bacterial DNA MiniPrep™ kit (Zymo Research, Irvine, CA, USA), bacterial genomic DNA was isolated from the actinomycetes following the manufacturer’s protocol. The 16S rRNA gene was amplified using 27f/1492r universal primer set. The PCR amplification was conducted in 20 µL reactions of 10 µL of Lucigen EconoTaq™ (Middleton, WI, USA) Plus Green 2X Master Mix, 1 mM of each forward and reverse primer, 1 µL of template DNA and nuclease-free water to makeup the final volume. The optimized PCR thermocycling conditions used was according to earlier report by Agunbiade [17]. The Thermoscientific thermal cycler PCR machine (Thermo Fisher Scientific Inc, MA, USA) was used to run the reaction. Amplification of the correct product was confirmed. The PCR products were electrophoresed in 1% (*w*/*v*) agarose gel, stained with gel red for confirming the amplification of the expected 16S rRNA gene fragments. Automated sequencing of 16S rRNA genes of the organism was done using the sequencer AB 3500× L genetic analyzer (Applied Biosystems). Sequencing reactions was performed according to the manufacturer’s protocol using Big Dye version 3.1 dye terminator cycle sequencing kit (Applied Biosystems, Foster City, CA, USA) with 27f and 1492r primers. In order to identify the bacteria, the sequences obtained were checked against the National Centre for the Biotechnology Information (NCBI) GenBank database using the BLASTn programme for their phylogenetic affiliations.

### 2.5. Purification of Bioflocculant

Extraction and purification of bioflocculant was performed as described by Chen et al. [18] and Piyo et al. [19]. After 72 h of fermentation (1 L of flask) under optimum conditions, the culture broth was centrifuged at 8000 g for 30 min at room temperature to remove the biomass cells. One volume of sterile distilled water was added to the supernatant phase and centrifuged at 8000 g for 15 min to remove insoluble substances. Thereafter, two volumes of ethanol was added to the supernatant, gently mixed and allow to stand for 12 h in the fridge. The obtained precipitate was vacuum-dried to obtain crude bioflocculant solution, to which one volume of a mixed solution of chloroform and n-butyl alcohol (5:2 v/v) was added. Subsequently, the mixture was stirred, poured into separating funnel, and allowed to stand for 12 h at room temperature. Finally, the supernatant was discarded and two volumes of ethanol were added to recover the precipitate and then lyophilized to obtain the purified bioflocculant. The crude product obtained was dissolved in water to yield a purified bioflocculants which was stored in the fridge prior further characterization and application.

### 2.6. Jar Test Determination of Bioflocculant Dosage

This was done according to the protocol of Lee [20] and Wang [21]. Different concentrations of powdered purified bioflocculant ranging from 0.1 to 1.0 mg/mL were prepared and their flocculating activities were measured against 4 g/L kaolin clay suspension.

### 2.7. Effect of pH and Cations on Bioflocculant Activity

The effect of pH on bioflocculant production was determined at the pH range of three to 11 using 1.0 M NaOH and 1.0 M HCl. Briefly, 2 mL of bioflocculant was used at different pH regime (three to 11) to evaluate the effect of pH on bioflocculant produced by the tested bacterial isolates. The flocculating activity was determined as earlier reported by Agunbiade [17]. In addition, the effect of Na^+^, K^+^, Mg^2+^, Mn^2+^, Al^3+^, and Fe^3+^ as representative of monovalent, divalent, and trivalent cations were evaluated on bioflocculant production. Calcium chloride, used as an enhancing agent, was replaced by metal salt solutions that were itemized above and flocculating activity was measured as previously described above.

### 2.8. Characterization of Purified Bioflocculants

#### 2.8.1. Chemical Composition Analyses

Protein content of the partially purified bioflocculant was measured by Bradford method using bovine serum albumin (BSA) as a standard [22]. Phenol sulfuric acid method using glucose as a standard solution was adopted in determining the total sugar content of the purified bioflocculant [23].

#### 2.8.2. Fourier Transform Infrared Spectroscopy (FTIR)

Fourier transform infrared spectroscopy was performed on the purified bioflocculants to determine the functional group present. This was achieved by pressing the bioflocculant homogeneously with KBr at the frequency range of 4000–650 cm^−1^.

#### 2.8.3. Thermogravimetric Analysis (TGA) and Thermal Stability

Thermo-gravimetric analyses of the purified bioflocculants were done using a thermo gravimetric analyzer (STA 449/C Jupiter, Netzsch, Germany Perkin Elmer TGA7, Waltham, MA, USA) over a temperature range of 20 to 600 °C at a heating rate of 10 °C/min under a constant flow of nitrogen gas. Furthermore, heat stability on the bioflocculant was performed using the method of Gong et al. [10] with little modification. After fermentation, two milliliters of the bioflocculant was heated at temperature regime of 30, 50, 70, and 90 °C, for 25 min. Afterwards, the supernatant broth was added to 100 mL of kaolin clay suspension with 3 mL of 1% CaCl_2_. Finally, flocculating efficiency was determined as earlier reported using Equation (1).

#### 2.8.4. Scanning Electron Microscopy (SEM) and Energy Dispersive X-ray Analysis

The purified bioflocculant samples were placed on a carbon coated stub and gold coated using Eiko IB3 ION coater. Scanning electron micrograph images of the bioflocculant was determined using a JEOL JSM-7800F FE-SEM coupled with an Oxford SDD X-Max EDS System employed for determining the elemental compositions of the partially purified bioflocculants.

### 2.9. Application of Purified Bioflocculant

#### 2.9.1. Treatment of Different Wastewaters by Bioflocculant Produced by *Terrabacter* sp.

Dairy, brewery, meat processed, river, and sewage wastewaters were collected from Gauteng and Eastern Free State Province of South Africa. Different physiological parameters such as pH, suspended solids, biological oxygen demand (BOD), turbidity, chemical oxygen demand (COD), and nitrate of the water sample were measured before and after treatment. Furthermore, dosage range of 0.1 to 1.0 mg/mL of partially purified bioflocculant produced by *Terrabacter* sp. and 3 mL of 1% (*w*/*v*) CaCl_2_ was added to flocculate 100 mL of each wastewater. A six padded stirrer was used to mix the suspension at 160 rpm for 2 min, and then at 40 rpm for 2 min. The treated samples were left to settle for 5 min and the supernatant was used for further physiological analyses. For comparison, organic synthesized flocculants (polyethylenimine and polyacrylamide) and inorganic flocculant (alum) were also tested. This experiment was conducted in triplicates and the turbidity, suspended solids, COD, and nitrates were measured using spectrophotometer DR 3800 and turbidimiter (HACH, USA). To assay for suspended solids, the contents were blended at 200 rpm for 1 min followed by blending at 60 rpm for another 5 min [24]. The removal efficiencies were thereafter calculated as:

(2) $$RE=\left[\frac{A_{o}-A}{A_{o}}\right]\times 100$$

where A_o_ and A are the initial and final values obtained before and after treatment respectively.

To assay for the BOD, 25 mL of raw and 50 mL of treated wastewater samples were added into BOD bottle and the bottles were filled up with BOD buffer (22.5 g MgSO_4_·7H_2_O, 27.5 g CaCl_2_, 0.25 g FeCl_3_·6H_2_O, and phosphate buffer solution). The BOD buffer was used as the initial working solution. The bottles were incubated at 20 °C for 5 days. The initial and final dissolved oxygen (DO) were measured after 15 min and 5 days, respectively using a HI5421 BOD Meter (Hanna, USA). The BOD and the percentage BOD removal efficiency were subsequently estimated using the equations:

(3) $$BOD=\frac{D_{1}-D_{2}}{P}$$

D_1_ = DO in diluted specimen after preparation

D_2_ = DO after five days

P = decimal fraction of specimen used

(4) $$RE\left(\%\right)=\frac{B_{1}-B_{2}}{B_{1}}\times 100$$

B_1_ = Untreated sample

B_2_ = Treated sample

#### 2.9.2. Assay for Removal of Heavy Metals by *Terrabacter* sp.

One hundred ml of dairy wastewater was treated with a bioflocculant dosage of 0.5 mg/mL and 3 mL of CaCl_2_ (1% *w*/*v*). The treated and untreated samples were analyzed at The Institute for Ground Water Studies, University of the Free State, South Africa, using inductible coupled plasma optical emission spectroscopy (ICP-OES) (Prodigy-7 Teledyne Leeman Labs, Mason, OH, USA) to validate the efficiency of the bioflocculant in removing suspected metals present in the wastewater samples.

#### 2.9.3. Statistical Analysis

Results were expressed as means ± standard deviation of three replicate determinations and were subjected to one-way analysis of variance (ANOVA) followed by Duncan multiple range tests to determine significant differences in all the parameters using SPSS 16.0 (IBM, Armonk, NY, USA). Values were considered statistically significant at *p*-values of less than 0.05.

## 3. Results

### 3.1. Isolation of Bioflocculant Producing Bacteria and Component Analysis

Four bacterial isolates that displayed promising flocculating activity against kaolin clay were selected for bioflocculant production. Strain SFD 11 which exhibited the highest flocculating efficiency of over 80% against kaolin suspensions was chosen for further studies. After 72 h of fermentation, 2.1 g of purified bioflocculant was recovered from 1 L of culture broth. Chemical analysis of the bioflocculant confirmed the proportion of total sugar and total protein content of the bioflocculant to be 71.6% and 1.7%, respectively, suggesting that the main component of the bioflocculant was polysaccharide. The 16S rDNA PCR yielded a product of expected size (approximately 1.5 kb). Basic local alignment search tool (BLAST) analysis of the nucleotide sequence of the 16S rDNA revealed the bacteria to have 98% similarity to *Terrabacter* sp. MUSC78T and the sequence was deposited in the Genbank as *Terrabacter* sp. with accession number KF682157.1.

### 3.2. Optimization of Flocculating Efficiency

#### 3.2.1. Effect of Bioflocculant Dosage

**Figure 1:**
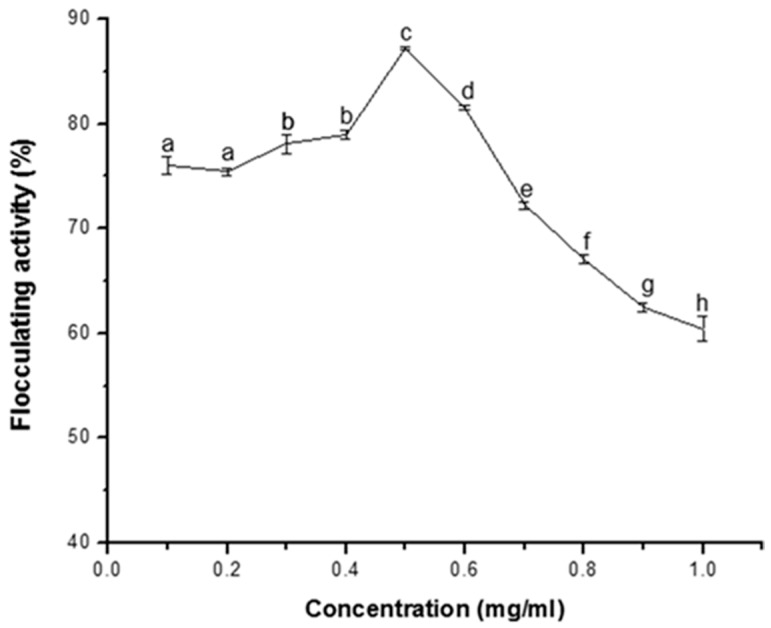
Effect of concentration on flocculating activity of purified bioflocculant produced by *Terrabacter* sp. Percentage flocculating activities with different alphabetic letters are not significantly different (*p* > 0.05).

The result of the flocculating efficiency of the purified bioflocculant over a dosage range 0 to 1.0 mg/mL is shown in Figure 1. Bioflocculant concentration of 0.1–0.4 mg/mL resulted in flocculating activity of over 75%. However, as the dosage was increased to 0.5 mg/mL, optimum flocculating activity of over 85% was observed, while further increase in concentration resulted in the decrease in flocculating activity (Figure 1).

#### 3.2.2. Effect of Temperature

**Figure 2:**
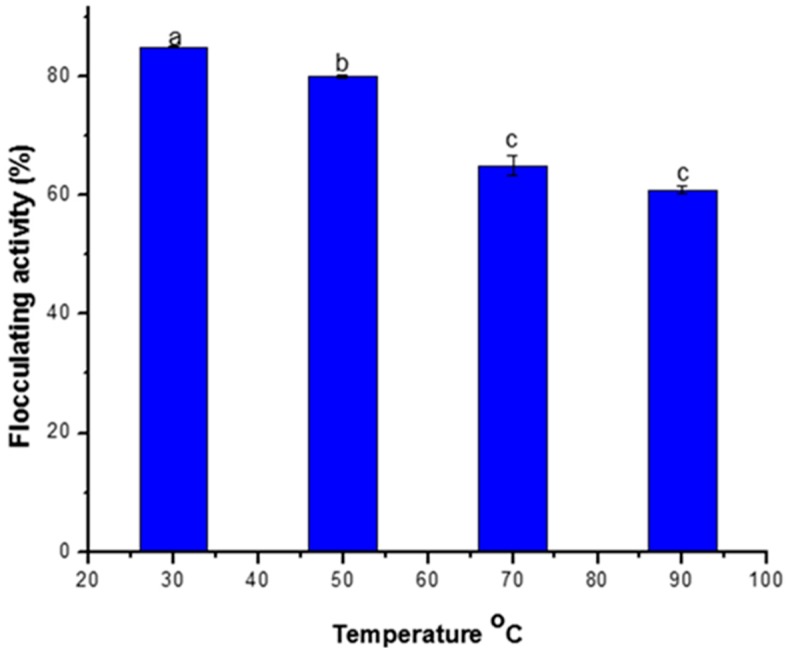
Thermal stability of bioflocculant produced by *Terrabacter* sp. Percentage flocculating activities with different alphabetic letters are significantly different (*p* < 0.05). Correct the axis so that it would reflect only the temperatures used.

The effect of temperature on the flocculating activity of the purified bioflocculant is presented in Figure 2. After heating the crude bioflocculant at 30 °C and 50 °C for 25 min, the bioflocculant maintained its flocculating activity at over 80%. However, flocculating activity slightly decreased to approximately 60% after heating the bioflocculant at 70 °C and 90 °C.

#### 3.2.3. Effect of Metal Ions

**Figure 3:**
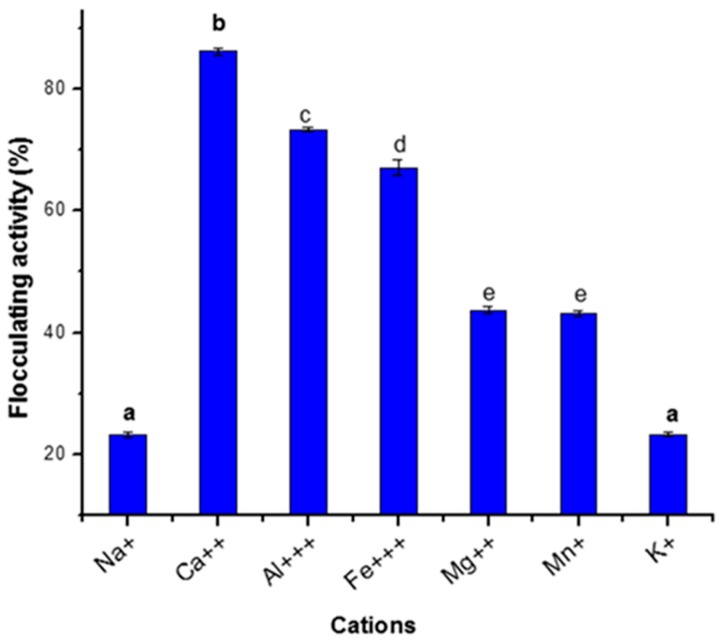
Effect of cations on flocculating activity. Percentage flocculating activities with different alphabetic letters are significantly different (*p* < 0.05).

Presented in Figure 3 is the observed influence of metal ion on the flocculating activity of the bioflocculant produced in this study. While Ca^2+^ resulted in optimum flocculation efficiency of 85%, the flocculation efficiency was however drastically reduced below 50% when Na^+^, Mg^2+^, Mn^2+^, and K^+^ were used as a representative of monovalent and divalent cations.

#### 3.2.4. Effect of pH

**Figure 4:**
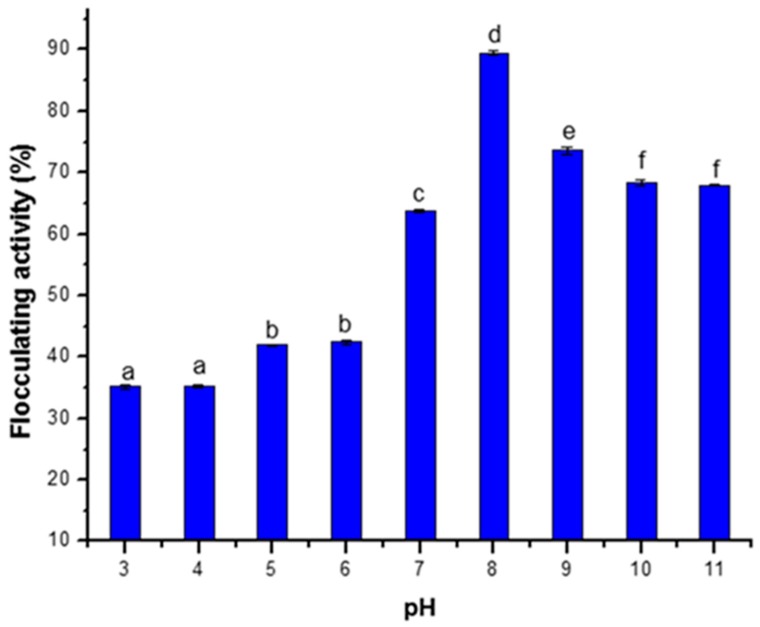
Effect of pH on bioflocculant production. Percentage flocculating activities with different alphabetic letters are significantly different (*p <* 0.05).

The characteristic efficiency of the bioflocculant produced in this study over different pH range revealed that both neutral and alkaline media supported flocculating activity of the bioflocculant, with maximum flocculating efficiency observed at pH 8 (Figure 4).

### 3.3. Functional Group Analysis

**Figure 5:**
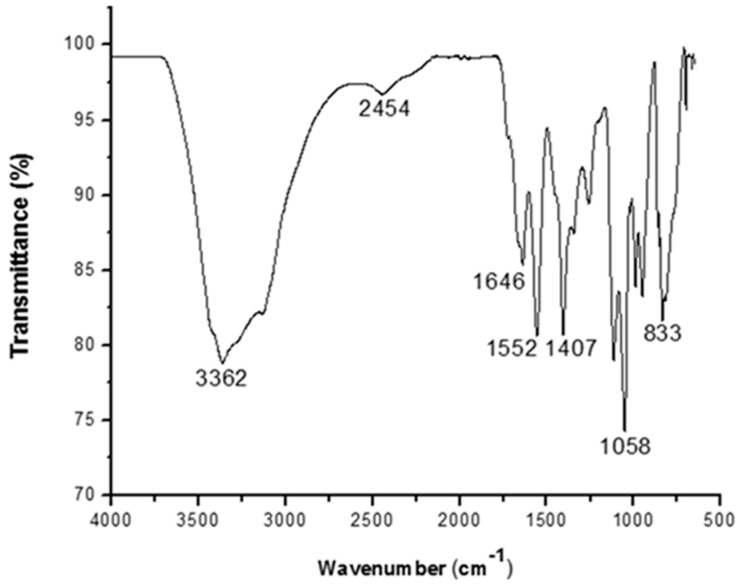
Fourier transform infrared (FTIR) spectrogram of purified bioflocculant produced by *Terrabacter* sp.

The Fourier transformer infrared spectrogram of the purified bioflocculant investigated in this study exhibiting diverse peaks from 4000 to 650 cm^−1^ is shown in Figure 5.

### 3.4. Thermogravimetric Analysis and Energy Dispersive X-ray and SEM Analyses

**Figure 6:**
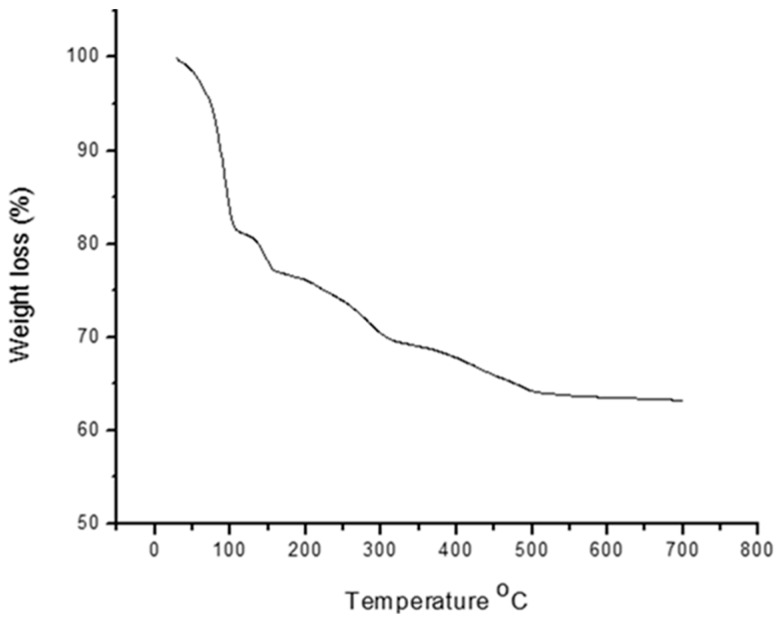
Thermogravimetric analysis of purified bioflocculant.

**Figure 7:**
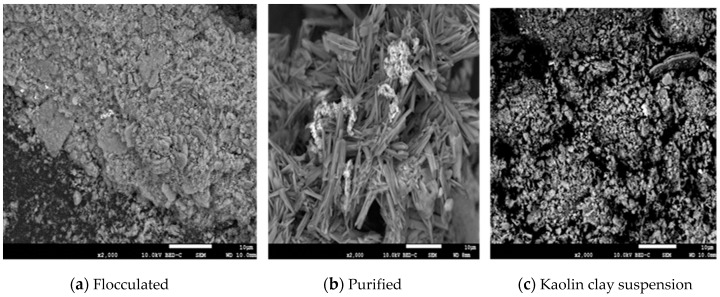
Scanning electron micrographs of (**a**) flocculating kaolin suspension (**b**) purified bioflocculant (**c**) Kaolin powder suspension.

The data obtained with respect to the thermogravimetric analysis and SEM of the test bioflocculant are presented in Figure 6 and Figure 7, respectively. There was about 18% loss in weight when the temperature was increased from 50 °C to 120 °C. Moreover, there was a gradual decomposition of the material at 120 °C to 500 °C and about 35% loss was observed. When the temperature was increased from 500 °C to 700 °C, the bioflocculant was stable and about 65% weight was retained (Figure 6).

The elemental analyses of the bioflocculant revealed that the weight fractions of the elements C, N, O, S, and P were 15.7%, 1.2% 51.2%, 0.1%, and 1.4%, respectively. The surface morphology of flocculated kaolin clay, purified bioflocculant, and kaolin clay suspension were observed under SEM as shown in Figure 7.

### 3.5. Application of Bioflocculant in Wasteswater Treatment

It is evident that the purified bioflocculant appeared to be wastewater specific (Table 1). In other words, bioflocculant produced by *Terrabacter* sp. could flocculate dairy wastewater as shown in Table 1. However, better flocculating efficiency was achieved against dairy wastewater and this validate the choice of conducting further analysis on dairy wastewater. On the other hand, the partial purified bioflocculant resulted in no flocculation of brewery, sewage, and meat processed wastewaters tested in this study. Sequel to the heavy metal analysis the physicochemical properties of the raw dairy wastewater are shown in Table 2. The comparison of experimental results obtained when the test microbial flocculant (SFD 11), PACL, PEI, and alum were used in treatment of dairy wastewater are shown in Table 3. Furthermore, the result of the heavy metal ion adsorption by the test bioflocculant (SFD11) revealed that it could remove 77.7% Fe, 74.8% Al, 61.9% Mn, and 57.6% Zn, respectively, at a bioflocculant dosage of 0.5 mg/mL (Table 4).

## 4. Discussion

Dosage requirement in flocculation is an important parameter that determined the optimum conditions for performance of flocculant in coagulation-flocculation technology. It has been documented that over dosage or insufficient dosage will result in poor performance of a flocculant [25]. In this study, the observed decrease in the flocculating activity beyond 0.5 mg/mL (Figure 1) could be attributed to obstruction of the site of adsorption which minimizes flocculation and formation of flocs [10,26,27], the optimum flocculating activity of over 85% exhibited by the bioflocculant could be said to be significant and compared well with the activity of the bioflocculant produced by *Bacillus* sp. AEMREG7 with flocculating activity of 92.6% at a concentration of 0.3 mg/mL [28].

According to Jing [29], thermal stability of bioflocculant is crucial to its commercial application. In the present study, the slight decrease in flocculating activity at higher temperature could be due to the breaking down of the polysaccharide chain which could result into slow formation of bridges with the kaolin particles. The heat stability displayed by the bioflocculant produced by *Terrabacter* sp. is consistent with the reported findings that flocculants rich in polysaccharide are more heat stable than those composed of mainly protein and nucleic acids [10,30] and could have a remarkable commercial value. Our result corroborates with the findings of Gong et al. [10] and Lu co-workers [28], where bioflocculant produced by *Serratia ficaria* and *Enterobacter aerogenes* were thermally stable and adjudged to be of high commercial value and better alternative to the synthetic flocculants.

Although, the addition of cations could help to neutralize negative charges on the bioflocculant and the suspended kaolin particles, shortens the distance between them, increase the initial adsorption of the bioflocculant onto the kaolin particle and thus leading to floc formation and sedimentation. However, the effect of cations may wary with flocculating activity of each bioflocculant [31]. In addition, metal ions have also been reported to inhibit flocculating activity or have no effect at all on the flocculating activity of bioflocculant [32]. In this study, the poor flocculating activity observed when Na^+^ and K^+^ were used might be due to the fact that monovalent cations could be responsible for the formation of bonds that are loose in structure [14]. This finding corroborates the report of Zhang co-workers [33] where the bioflocculant produced by *Proteus mirabilis* was enhanced with the addition of Ca^2+^ while, KCl resulted in little or no flocculating activity. In addition, bioflocculant produced by *Bacillus subtilis* was stimulated in the presence of Al^3+^ and Fe^3+^ [34].

The pH of a solution plays a vital role in flocculating efficiency and stability of flocs [35,36]. The most pronounced activity of the tested bioflocculant at pH 8 in this study agrees with the report of Wan et al. [34], where *Solibacillus silvestris* was grown in pH range of 7–9 and optimum flocculating efficiency of micro algal cells was obtained at pH 8. However, the initial pH requirement for optimum bioflocculant production differs with different bacterial strains. In another study, it was observed that the highest flocculation activity was attained at a pH value above 12 for bioflocculant produced by *Arthrobacter* sp. B4, [37]. In addition, alkaline pH range of 7–12 was favorable for bioflocculant produced by *Bacillus megaterium* and maximum yield of bioflocculant was obtained at pH 9, while it was inhibited in an acidic medium [38].

From the FTIR analysis, the broad intense peak at 3362 cm^−1^ suggest the presence of hydroxyl and amino groups in the purified bioflocculant and the small band at 2454 cm^−1^ could be attributed to C-H stretching. The peak at 1646 cm^−1^ and 1552 cm^−1^ is characteristics of carbonyl group stretching in an amide group [31,39]. The peak at 1058 cm^−1^ which corresponds to C-O stretching vibration in alcohols suggested the presence of hydroxyl group in the bioflocculant [8]. The peak at 833 cm^−1^ is characteristics of sugar derivatives. The FTIR analysis demonstrated that the presence of carboxyl, hydroxyl, and the sugar derivative confirmed that the main component of the bioflocculant is a polysaccharide and this observation is consistent with previous studies [6,10].

The observed initial weight loss of the tested bioflocculant as revealed in the thermogravimetric analysis in this study could be attributed to the moisture content in the purified bioflocculant [40]. Our result is in agreement with previous submission [41] ascribing the moisture content loss to the availability of carbonyl and hydroxyl group in the molecular structure of the purified bioflocculant and that it was rich in polysaccharides.

The interaction of kaolin clay suspension with the purified bioflocculant resulted to rapid aggregation of kaolin clay forming larger flocs. Thus, suggesting that flocculation might be achieved through bridging. The rod like clumpy structure of the purified bioflocculant serves as the site of attachment for cations and suspended kaolin particles [42].

Water has been a key processing medium in dairy industries and it has been used in cleaning, heating, cooling, and floor washing. Hence, there is a great demand of water in dairy industries and the need for suitable technology to recycle the wastewater which are being used in the plant. The concept of coagulation-flocculation technology is the best reliable treatment method of reducing suspended and colloidal particles that are responsible for turbidity of the wastewater. It is also employed in reducing organic matter that is responsible for the BOD and COD content of wastewater. Dairy wastewater is free of most toxic chemicals that are listed under Environmental Protection Agency’s (EPA) toxic release inventory. However, it contains high concentration of dissolved organic materials like whey proteins, lactose, fats, and minerals [43]. The performance of flocculants is highly dependent on dosage concentration [44]. In this study, the dosage concentration for each flocculants (0.1–1.0 mg/mL) was used in the treatment of dairy wastewater and each flocculants attained its maximum efficiency with respect to BOD removal, COD reduction, turbidity removal, and flocculation efficiency at different dosage concentration as reported in Table 3. After the interaction of the flocculants with the wastewater, turbidity reduction for SFD 11, PACl, PEI, and alum was 89.7, 87.2, 52.1 and 43% respectively with BOD and COD removal efficiencies of (63.3 and 54.1%), (60.9 and 43.3%), (46.0 and 36.0%), and (33.4 and 20.9%), respectively. Interestingly, the bioflocculant exhibited better flocculating activity by removing suspended solids and nitrate at efficiencies of 66.6 and 75.6%, respectively. In addition, this bioflocculant had higher removal rate than the other tested flocculants for BOD and COD. The exceptional turbidity removal could be as a result of polymer-floc interaction of the microbial flocculant that led to increase in the conglomeration of suspended particles. Moreover, the significant flocculating efficiency exhibited could be attributed to the presence of carboxyl and hydroxyl group in the functional group of the bioflocculant (Figure 5). The presence of carboxyl and hydroxyl group has higher adsorption forces that facilitates the process of aggregation in floc formation. Hence, they may be the preferred group for floc formation [45]. Our finding is consistent with results on the bioflocculant produced by *Serratia ficaria and Bacillus lichenifromis* when compared with other conventional flocculants [10].

Bioflocculants can be an effective biotechnological tool for the removal of toxic metals from water bodies and polluted industrial effluents. The rate and mechanisms of metal uptake by microbial flocculants rely on initial metal concentration, bioflocculant dosage, pH, temperature, and the conformational polymer type with adsorbed ions [42,46,47]. Cations enhanced the flocculating efficiency of the bioflocculant produced by *Terrabacter* sp. by neutralizing the negatively charged functional groups on the bioflocculant and suspended particles in dairy wastewater. Thus, enhancing the adsorption of the bioflocculant to the suspended particles in the wastewater. In this study, the highest adsorption activity exhibited by Fe^3+^ and Al^3+^ could be attributed to higher ionic valence which allows easy binding of the metals to the bioflocculant. The adsorption of heavy metal ions by bioflocculants producing acidic polysaccharides as their main backbone has been reported for bioflocculants [10,34,48].

## 5. Conclusions

This study shows that *Terrabacter* sp. isolated from Sterkfontein Dam is a bioflocculant that possesses the characteristics of an ideal flocculant needed in the treatment of industrial wastewater. The bioflocculant was thermally stable and chemical analysis confirmed that the main backbone is a polysaccharide. SFD 11 exhibited effective flocculation ability and was able to significantly remove BOD, COD, turbidity, and heavy metal ions in dairy wastewater. Hence, this study suggests the potential usage of the bioflocculant SFD 11 as an alternative means in wastewater treatment and other biotechnological processes.

## Figures and Tables

**Table 1:** Screening of bioflocculant produced by *Terrabacter* sp. in the treatment of river, dairy, brewery, meat processed, and sewage wastewaters.

Bioflocculant Dosage (mg/mL)	(%) Flocculating Activity				
	River	Dairy	Sewage	Meat Processed	Brewery
0.1	18.1 ± 1.5	61.0 ± 2.3	-	-	-
0.2	22.6 ± 1.9	61.7 ± 0.9	-	-	-
0.3	22.8 ± 0.2	63.0 ± 0.7	-	-	-
0.4	24.2 ± 2.9	69.8 ± 0.2	-	-	-
0.5	17.5 ± 0.3	85.1 ± 0.1	-	-	-
0.6	17.1 ± 1.6	79.4 ± 0.1	-	-	-
0.7	15.9 ± 0.8	74.0 ± 0.9	-	-	-
0.8	08.3 ± 2.4	73.5 ± 1.6	-	-	-
0.9	07.6 ± 1.6	71.7 ± 0.7	-	-	-
1.0	07.7 ± 1.3	70.8 ± 1.9	-	-	-

(-) denotes no flocculating activity.

**Table 2:** Physicochemical properties of untreated dairy, river, meat processed, sewage, and brewery wastewaters.

Wastewater	pH	Turbidity (NTU)	COD (mg/L)	Nitrate (mg/L)	BOD (mg/L)	SS (mg/L)
Dairy	7.21 ± 1.4	907 ± 0.9	1758 ± 2.1	5.24 ±1.3	623 ± 0.3	528 ± 2.4
River	7.10 ± 0.9	123 ± 1.6	170 ± 0.3	2.40± 0.6	34.0 ± 1.6	186 ± 1.1
Meat	8.90 ± 1.5	238 ± 1.9	356 ± 0.5	6.70 ± 0.4	305 ± 0.8	224 ± 0.1
Sewage	7.73 ± 0.2	128 ± 1.1	1360 ± 1.7	8.40 ± 0.3	49.2 ± 0.2	201 ± 1.4
Brewery	6.28 ± 1.6	442 ± 2.3	4033 ± 2.4	9.60 ± 1.8	1703 ± 2.1	461 ± 0.9

NTU: Nephelometric turbidity units; COD: Chemical oxygen demand; BOD: Biological oxygen demand.

**Table 3:** Comparison of the flocculating activity of *Terrabacter* sp. bioflocculant with organic and inorganic flocculants for diary wastewater.

Flocculant	Dosage (mg/mL)	BOD Removal (%)	COD Removal (%)	Turbidity Removal (%)	SS (mg/L)	F/A	Nitrate (mg/L)
SFD 11	0.5	63.3 ± 0.4	54.1 ± 0.5	89.7 ± 0.6	66.6 ±1.2	85.1 ± 0.1	75.6 ± 0.4
PAC	0.3	60.9 ± 0.7	43.3 ± 0.8	87.2 ± 1.1	71.2 ± 0.5	79.0 ± 0.6	68.1 ± 2.9
PEI	0.7	46.0 ± 1.4	36.0 ± 0.4	52.1 ± 0.5	43.6 ±1.4	56.0 ± 1.0	49.3 ± 1.2
Alum	1.0	33.4 ± 1.3	20.9 ± 0.6	43.0 ± 1.5	50.2 ± 0.9	38.1 ± 0.5	51.8 ± 0.8

The Values are expressed as means ± SD of triplicate determinations. SD: Standard deviation; PAC: Polyaluminum chloride; PEI: Polyethylenime; F/A: Flocculating activity; COD: Chemical oxygen demand; BOD: Biological oxygen demand; SFD11: *Terrabacter* sp.

**Table 4:** Application of bioflocculant produced by *Terrabacter* sp. in heavy metals removal from diary wastewater.

Metals	Treated Sample	Untreated Sample	(%) Removal
Aluminum	0.029	0.115	77.7
Manganese	0.008	0.021	74.8
Zinc	0.042	0.099	61.9
Iron	0.029	0.130	57.6
